# Nutrient Intake and Risk Factors for Metabolic Syndrome in Christian Orthodox Church Religious Fasters

**DOI:** 10.3390/nu15112468

**Published:** 2023-05-25

**Authors:** Anna Kokkinopoulou, Niki Katsiki, Ioannis Pagkalos, Nikolaos E. Rodopaios, Alexandra-Aikaterini Koulouri, Eleni Vasara, Sousana K. Papadopoulou, Petros Skepastianos, Emmanouil Dermitzakis, Maria Hassapidou, Anthony G. Kafatos

**Affiliations:** 1 Department of Preventive Medicine and Nutrition Unit, School of Medicine, University of Crete, 71003 Crete, Greece; 2 Department of Nutritional Sciences and Dietetics, International Hellenic University, 57400 Thessaloniki, Greecemnhas@ihu.gr (M.H.); 3 School of Medicine, European University Cyprus, Nicosia 2404, Cyprus; 4 Laboratory of Animal Physiology, Department of Zoology, School of Biology, Aristotle University of Thessaloniki, 54124 Thessaloniki, Greece; 5 Department of Medical Laboratory Studies, International Hellenic University, 57400 Thessaloniki, Greece; pskep@otenet.gr; 6 Department of Genetic Medicine and Development, Faculty of Medicine, University of Geneva, 1211 Geneva, Switzerland

**Keywords:** nutrient intake, metabolic syndrome, protein, fat, cholesterol, Christian Orthodox Church fasting

## Abstract

Objective: Studies regarding health effects of religious fasting have been increased during the last decade. Our aim was to investigate the impact of adherence to the periodic Christian Orthodox Church (COC) fasting on nutrient intake, body composition, and risk factors for metabolic syndrome (MetS). Methods: Four-hundred individuals aged 42.6 ± 17.0 years participated in this cross-sectional study. Two-hundred subjects followed the COC fasting since childhood or at least the last twelve consecutive years, and two-hundred subjects did not follow the COC fasting regimes or any other restrictive dietary pattern. Socioeconomic data, lifestyle habits, and physical activity data were collected. Nutritional assessment was performed via two 24 h recalls and a food frequency questionnaire. Anthropometric data and biochemical parameters were also measured. Results: Fasters had a significantly lower daily intake of calories (1547 vs. 1662 kcals, *p* = 0.009), protein (52 vs. 59 g, *p* = 0.001), fat (82 vs. 89 g, *p* = 0.012), and cholesterol (147 vs. 178 g, *p* = 0.001) compared with non-fasters. Furthermore, fasters reported a healthier way of living, with lower rates of smoking and alcohol consumption (*p* < 0.001 and 0.002, respectively). Insulin and magnesium levels were significantly higher, whereas levels of urea, transaminases, glucose, and phosphorus were significantly lower, as was DBP in fasters versus non-fasters. Furthermore, MetS prevalence was non-significantly higher in non-faster compared with fasters. Conclusion: During a non-fasting period, individuals following the COC fasting recommendations reported lower intake of calories, protein, fat, and cholesterol compared with non-fasters. Fasters tended to have a healthier lifestyle pattern and a lower risk for MetS versus non-fasters. Some biochemical parameters also significantly differed between the two study groups. Further research is warranted to establish the long-term clinical impact of these findings.

## 1. Introduction

The well-known dictum “Let food be thy medicine and medicine be thy food” by Hippocrates, the most famous physician of the ancient Greece, was first recorded around 400BC. This was one of the oldest pieces of evidence that connects diet with disease prevention [1,2]. Adherence to the Mediterranean diet principles has been associated with a low risk of obesity and metabolic syndrome (MetS) development [3], as also supported by a recent meta-analysis showing that the greater the adherence to the Mediterranean diet, the higher the reversion of MetS and/or its components [4].

The fundamentals of the Mediterranean diet were first studied by Ancel Keys in the Seven Countries’ Study (SCS), leading to the recognition of this diet as the optimal one in the 1960s [4,5]. In brief, the Greek participants, who were inhabitants of Crete, had the highest rates in longevity, in comparison to individuals from other countries [6,7]. When the demographics of the 686 middle-aged healthy Cretan men were studied in further detail, it was shown that 60% of them mentioned a strict adherence to the Christian Orthodox Church (COC) fasting recommendations [7,8,9]. COC fasting covers in total 180–200 days annually, i.e., almost every Wednesday and Friday, 40 days before Christmas, 48 days before Easter, 15 days before the Assumption, and approximately 30 days prior to the feast of the Holy Apostles. During these periods, a plant-based diet is followed, ranging from pescatarian diets (fish, seafood) that include snails, to vegan diets based on a religious calendar [3]. Total abstinence from meat, dairy products, and eggs is compulsory, while the consumption of fish is strictly allowed only on limited days during the fast. Overall, for the majority of the fasting periods, the diet is a vegetarian diet with the addition of seafood and snails. Given that 50–60% of the year is dedicated to fasting, the traditional Mediterranean diet of Greece strongly mimics those fasting dietary guidelines [3].

Although the health effects of the Mediterranean diet and its recognition as one of the healthiest diet patterns in the world are well documented [4,10,11,12], the health effects of the COC fasting have been investigated only during the last decades, with an increased interest being documented in scientific databases. Little is known about the impact of COC fasting on nutrient intake, body composition, blood lipids, and MetS presence. MetS, a major worldwide healthcare issue, is a cluster of heart disease risk factors, which includes central obesity (abdominal obesity), hyperglycemia, hypertriglyceridemia, low high-density lipoprotein (HDL) cholesterol, and hypertension [13]. According to the harmonized criteria, MetS is defined when three or more of the criteria are met: (i) waist circumference ≥ 102 cm for males and ≥88 cm for females (or according to each country cut-offs), (ii) fasting glucose ≥ 100 mg/dL or on antihyperglycemic drug treatment, (iii) triglycerides ≥ 150 mg/dL or on hypertriglyceridemia drug treatment, (iv) HDL cholesterol < 40 mg/dL for males and <50 mg/dL for females or on drug treatment, and (v) SBP (SBP) ≥ 130 mmHg or DBP (DBP) ≥ 85 mmHg or on antihypertensive drug treatment [14].

According to a recent review, adherence to the COC fasting recommendations could be beneficial for the lipid profile and body composition, without leading to iron deficiency or decreased bone mineral density; however, the evidence is still limited [3]. Weight changes, especially weight loss, are common during the longer fasting periods [15,16,17,18,19,20], and it is often linked to fat loss [21]. However, it is unclear as to whether those changes in body weight and composition are maintained after the fasting period or not.

Therefore, the aim of the current study was to explore the relationship between COC fasting adherence, nutrient intake, body composition, and MetS among individuals with long-standing (since childhood or for more than twelve consecutive years) adherence to the COC dietary recommendations as compared with individuals that do not follow any dietary restrictions.

## 2. Methods

### 2.1. Study Design and Study Population

This cross-sectional study was performed in the Alexander Technological Educational Institute of Thessaloniki, Greece. The study protocol was approved by the Bioethics Committee of the Institute. The researchers explained the purpose of the study to eligible participants. All participants read the participant information sheet, understood the purposes of the study, and provided their written informed consent to participate in the study, and they were free to withdraw at any time without repercussions. Participants were recruited on a voluntary basis from Thessaloniki, the sub-capital of Greece, as a response to email invitations sent to the Aristotle University of Thessaloniki, the Alexander Technological Educational Institute of Thessaloniki, churches, and monasteries, all located in Thessaloniki. A closed-ended questionnaire regarding diet status was used to determine participation in the study, with those qualified as fasters declaring full adherence to the COC fasting regimes since their childhood or for at least the last twelve consecutive years. The aim of the study was to focus on the long-term effects of the COC fasting on health, and therefore people following the specific diet pattern since childhood or for a period of at least 12 consecutive years were needed. On the other hand, participants who qualified as non-fasters stated that they did not follow any specific diet, such as the lacto-ovo-vegetarian or DASH diet, and did not avoid any specific food item for any medical or other reason. Inclusion criteria were also (i) being an adult (i.e., >18 years old); (ii) being able to provide written, informed consent; (iii) being able to participate in all anthropometric measurements at a scheduled timepoint; (iv) not being currently pregnant of breastfeeding; and (v) being in good general health with no long-term co-morbidities (e.g., diabetes mellitus).

**Figure 1:**
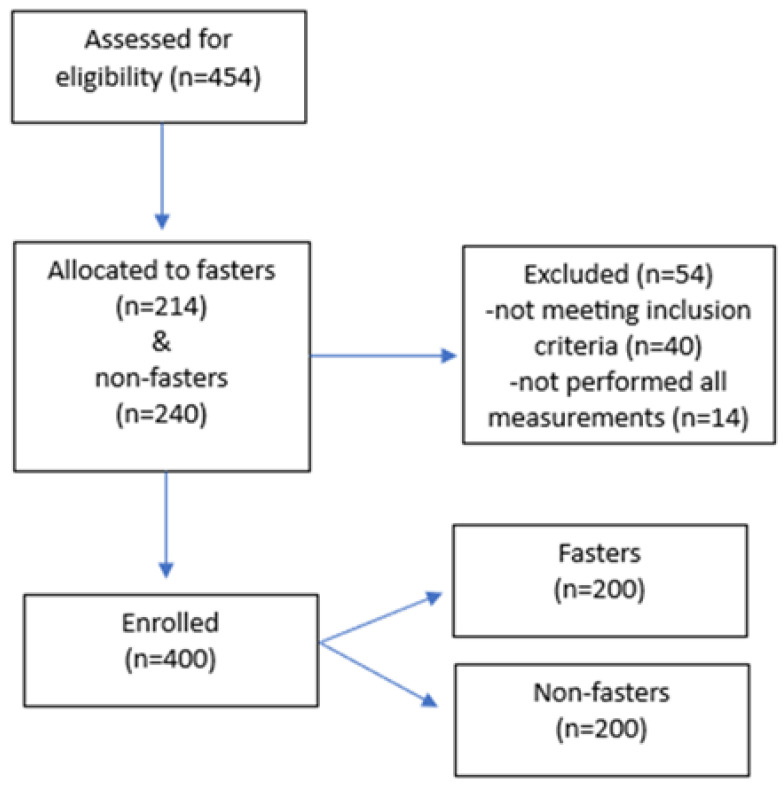
Participant flow diagram of the study.

Overall, 454 individuals consented to participate in the study. Forty individuals were excluded, since inclusion criteria were not met, and fourteen more were not able to follow the scheduled appointments to complete all anthropometric measurements. Therefore, 400 participants (143 men and 257 women) with a mean age of 42.6 ± 17.0 years participated in the study, as seen in the participants’ flow diagram (Figure 1). Participants were equally divided between fasters and non-fasters. In more details, 200 individuals (69 men and 131 women), mean age 43.3 ± 16.6 years, fasted regularly according to the COC fasting regime since their childhood or for at least the last twelve consecutive years, and they formed the group defined as fasters. Two-hundred individuals (74 men and 126 women) with a mean age of 41.9 ± 17.3 years were the non-fasters, since they did not fast and/or follow any other restrictive dietary pattern.

### 2.2. Socioeconomic, Lifestyle, and Physical Activity Habits

All participants completed a validated questionnaire regarding their socioeconomic status (educational level, marital status) and lifestyle habits, with questions including smoking status, alcohol consumption, time spending using the computer, time watching television, and time sleeping, among others [22]. Closed questions were used for educational level (answers provided: primary education, secondary education, tertiary education, and MSc/PhD) and marital status (with answers: single, married/living together, divorced, and widowed). The habit of smoking was screened by selecting one of three categories: yes, no—never, and no—quit smoking, and the habit of drinking alcohol by selecting one of two categories: yes and no. Questions regarding time sleeping during a typical day and night, time using the computer, and time watching TV during a typical day were assessed through open-ended questions, where participants reported the exact time spending doing these activities. Furthermore, the frequency of physical activity per week as well as its duration for each session was reported with open-ended questions, and the physical activity status was recorded via closed questions (with answers: never/rarely (extremely low), <2 times per week (low), 2–3 times per week (moderate), 3–5 times per week (high), everyday (extremely high)).

### 2.3. Anthropometric Measurements

Anthropometric measurements were performed by a trained dietitian. Prior to the scheduled appointment, all participants were informed to abstain from any food or liquid intake for 3 h prior to measurements, as well as any form of physical activity for 24 h.

Height was measured to the nearest 0.5 cm using a stadiometer (HR-001, ΤAΝΙΤA, Manchester, UK), with participants asked to wear no shoes, with light clothing, having shoulders relaxed, legs straight, arms at sides, and standing with their back against the stadiometer (Frankfort Plane position). Body weight was measured to the nearest 0.1 kg with the use of a calibrated digital scale (SECA 876, SECA, Hamburg, Germany), with participants asked to remove outer clothing, shoes, and anything in their pockets, standing in the middle of the scale with feet slightly apart and arms relaxed. Body mass index (BMI) was calculated as body weight divided by the square of height (kg/m^2^). Waist circumference was measured with a stretch-resistant tape over the naked skin or underwear, after a normal expiration, with arms relaxed at the sides under the midline of the participant’s armpit, at the midpoint between the lower part of the last rib and the top of the hip. Waist circumference was measured with an accuracy of 0.1 cm, two times, and an average value was recorded. Hip circumference was measured with the same tape over the naked skin or underwear, with the arms relaxed at the sides and at the maximum circumference over the hips. Hip circumference was also measured twice with an accuracy of 0.1 cm, and an average value was recorded.

For the measurement of body composition (i.e., body fat, muscle mass, and total body water), the Bioelectrical Impedance Analysis (BIA) method was used, with a calibrated Bioimpedance Analyser (BODYSTAT 1500, BODYSTAT, Douglas, Isle of Man). Participants were asked to lie in an examination table and remove all jewelry from their bodies. In order to reduce any skin-to-skin contact errors, participants were asked to have their arms separated from the trunk, with legs apart from each other.

### 2.4. Nutritional Assessment

A combination of dietary assessment methods was used in this cross-sectional study. Three interviewer-administered 24 h diet recalls were collected, in order to capture detailed information about all foods and beverages consumed during the study week. Food records were for two weekdays and a weekend day. A food frequency questionnaire (FFQ) validated in the Greek population was used to estimate frequency of consumption of 114 different foods and beverages in a month [22]. Participants could select one of six categories that reported monthly consumption of food or beverage: never, once to three times per month, once to twice per week, three to six times per week, once per day, and equal or more than two times per day. The FFQ was answered with the supervision of a trained dietitian. The accuracy of portion sizes was assisted by the use of a validated food atlas with food portion sizes photographed in plates [23], the use of food replicas and models, and household measures such as cups and plates. For the analysis of food records, the Food Processor nutrition analysis software (version 11.7) (ESHA, Salem, OR, USA) was used, in which Greek recipes from the Greek food composition tables were added.

Nutritional behavior was studied through a validated questionnaire [22], which included questions about eating habits, cooking methods, mindful eating, and supplement and table salt use. More specifically, the questionnaire included questions on the frequency of breakfast consumption (daily to never), number of eating occasions (1–5 per day), addition of table salt (yes/no), and preferred cooking method (boiling, roasting, frying). Mindful eating was assessed through a question on the mode of eating (slow, quick, anxious eating) and a question on whether the participants consumed their meals alone, with company, or alone in front of a TV/screen. Supplement use was screened through a yes/no question, and in the case of a positive answer, participants were asked to report which supplements they used. Lastly, the use of table salt apart from the cooking process was assessed through a question that received yes/no answers.

### 2.5. Blood Pressure and Blood Analysis

Blood pressure (BP) was measured with an electronic BP monitor while the individuals were relaxed in a seated position for 3–5 min. Two measurements were performed, separated by 2 min, and the average of the measurements was recorded, following the technique of the National Institute for Health Care Excellence (NICE) and European Society of Hypertension (ESH) guidelines [24]. Finally, 6 mL of venous blood was collected, with participants in a seated position and after a 12 h fast, for biochemical analysis. Blood was drawn into EDTA sample tubes, and the serum was separated by centrifugation and then stored in the freezer at −80 °C. Analysis was performed at a certified lab that was involved in a quality control program.

### 2.6. Statistical Analysis

The normality of distribution of continuous data was assessed with the Kolmogorov–Smirnov test and histogram charts. Continuous data are shown as means with standard deviation (SD) and categorical variables as relative frequencies with percentages. Comparisons between the two diet groups (fasters versus non-fasters) were performed with the chi-squared test, while Student’s *t*-test and one-way analysis of variance (ANOVA) were used to test for differences in continuous variables among two or more groups, respectively. The SPSS version 21 software (SPSS, Chicago, IL, USA) was used for all presented statistical analyses, with statistical significance set at two-sided *p* = 0.05.

## 3. Results

Overall, fasters (*n* = 200) followed the COC fasting regime for a mean period of 25.3 ± 15.4 years, starting since their childhood or for the last twelve consecutive years. Fasters were 69 men and 131 women, with a mean age of 43.3 ± 16.7 years, mean body weight 74.3 ± 15.4 kg, and mean BMI 26.7 ± 4.7 kg/m^2^. Moreover, mean body fat was 30.8 ± 9.7%, mean waist circumference was 89.3 ± 14.3 cm, and hip circumference was 99.2 ± 9.3 cm. On the other hand, non-fasters were 200 individuals with a mean age of 41.9 ± 17.3 years, mean weight 73.6 ± 15.5 kg, and mean BMI 26.0 ± 4.4 kg/m^2^. Furthermore, mean body fat was 30.2 ± 8.8%, mean waist circumference was 88.5 ± 13.1 cm, and hip circumference was 98.4 ± 7.3 cm. There were no significant differences in anthropometric measurements between the two diet groups (Table 1). In contrast, DBP was significantly higher in non-fasters (*p* = 0.003).

The distribution of sexes and age was similar among fasters and non-fasters (*p* = 0.60 and 0.40, respectively). As noticed in the results, we had 69 and 74 males as fasters and non-fasters respectively, and in terms of females, the representation was 131 and 126 for fasters and non-fasters, respectively. In terms of age, fasters had a mean age of 43.3 ± 16.7 years, with a minimum age of 18 and maximum of 77.6, and non-fasters had a mean age of 41.9 ± 17.3 years, with a minimum age of 18 and maximum of 77.3. No differences were observed in the education and marital status between the two groups (*p* = 0.17 and 0.67, respectively). As far as lifestyle characteristics are concerned, fasters were more likely to be non/ex-smokers (*p* < 0.001). In more detail, 91.5% of fasters did not smoke compared with 29% of non-fasters, while 5.5% of fasters smoked compared with 71% of non-fasters. In terms of physical activity levels, no significant differences were noted (*p* = 0.06).

Fasters were also more likely to report a regular way of eating compared with non-fasters (*p* = 0.004), as well as a limited consumption of diet products (*p* = 0.002). In addition, fasters spent significantly less hours in sleeping/napping during the day (*p* = 0.008), as well as less time watching TV (*p* = 0.000), using mobiles and/or PCs (*p* = 0.043), and engaging in free-time workouts (*p* = 0.021). Demographic characteristics are shown in Table 2 and in Supplementary Materials Table S1.

According to the mean intake of the three 24 h recalls, fasters had a significantly lower mean energy intake compared with non-fasters (1547 ± 389 vs. 1662 ± 480 kcal, respectively, *p* = 0.009). Similarly, a significantly lower mean intake was observed in fasters for protein (*p* = 0.001), carbohydrate (*p* < 0.001), total sugars (*p* < 0.001), other carbohydrates (*p* = 0.036), fat (*p* = 0.012), saturated fat (*p* = 0.001), polyunsaturated fat (*p* = 0.009), total cholesterol (*p* = 0.001), vitamin A (*p* = 0.004), retinol (*p* = 0.008), vitamin B12 (*p* < 0.001), vitamin C (*p* = 0.015), folic acid (*p* = 0.004), pantothenic acid (*p* < 0.001), calcium (*p* < 0.001), phosphorus (*p* < 0.001), zinc (*p* < 0.001), and alcohol (*p* = 0.002), in comparison to non-fasters (Table 3).

Non-fasters had significantly higher levels of urea (*p* = 0.049), SGOT (*p* = 0.016), SGPT (*p* = 0.008), phosphorus (*p* < 0.001), and glucose (*p* = 0.018), and significantly lower levels of insulin (*p* = 0.036) and magnesium (*p* < 0.001) compared with fasters. Results can be found in Table 4 for more details.

With regard to MetS components and prevalence, the non-fasting group had more frequently elevated fasting blood glucose (*p* = 0.013) and BP (*p* < 0.001), while no significant difference was observed in elevated waist circumference and triglycerides (*p* = 0.18 and 0.39, respectively) and decreased HDL cholesterol levels (*p* = 0.51). Although more non-fasters had MetS compared with fasters (21.0% vs. 15.5%, *p* = 0.15), the difference was not statistically significant (Table 5).

In the group of fasters, gender analysis showed that there were significant differences in some anthropometric measurements and body composition. In more detail, male fasters had a higher mean weight (83.5 ± 16.4 vs. 69.5 ± 12.4 kg, *p* < 0.001), height (1.74 ± 0.06 vs. 1.62 ± 0.05 m, *p* < 0.001), fat free mass (62.5 ± 7.3 vs. 44.6 ± 3.6 kg, *p* < 0.001), waist circumference (92.5 ± 14.2 vs. 87.6 ±14.1 cm, *p* = 0.020), and waist-to-hip ratio (0.94 ± 0.10 vs. 0.87 ± 0.11, *p* < 0.001), whereas women had higher mean body fat (34.59 ± 8.3 vs. 21.0 ± 10.9%, *p* < 0.001). In regards to BMI, no difference was seen (*p* = 0.262), with both genders having similar mean values (27.2 ± 4.7 in men vs. 26.5 ± 4.7 kg/m^2^ in women). Men had significantly higher SBP (130.88 ± 12.0 in men vs. 123.5 ± 13.5 mmHg in women, *p* < 0.001) and DBP (81.2 ± 8.4 in men vs. 75.6 ± 8.3 in women, *p* < 0.001).

Similarly, male fasters had significantly greater mean intake of energy (1677 ± 413 vs. 1478 ± 358 kcal, *p* < 0.001), protein (57.8 ± 21.6 vs. 49.3 ± 16.7 g, *p* = 0.002), other carbohydrates (76.39 ± 25.5 vs. 66.0 ± 25.7 g, *p* = 0.007), fat (89.8 ± 28.0 vs. 77.2 ± 26.1 g, *p* = 0.002), polyunsaturated fat (9.5 ± 3.3 vs. 8.5 ± 3.3 g, *p* = 0.003), monounsaturated fat (45.2 ± 17.0 vs. 40.3 ± 15.4 g, *p* = 0.003), and water (754.3 ± 223.9 vs. 687.1 ± 227.9 mL, *p* = 0.048). Men also had higher levels of creatinine (1.02 ± 0.1 vs. 0.88 ± 0.1 mg/dL, *p* < 0.001), uric acid (5.3 ± 1.2 vs. 3.9 ± 1.07 mg/dL, *p* < 0.001), albumin (4.4 ± 0.2 vs. 4.2 ± 0.2 g/dL, *p* < 0.001), γ-GT (24.4 ± 15.9 vs. 17.1 ± 11.1 U/L, *p* < 0.001), iron (107.3 ± 39.6 vs. 95.4 ± 36.3 μg/dL, *p* = 0.034), SGOT (15 ± 9 vs. 12 ± 6 U/L, *p* = 0.002), and SGPT (10.1 ± 2.1 vs. 7.3 ± 3 U/L, *p* = 0.008) compared with women, who had higher mean values of HDL cholesterol (56.3 ± 16.4 vs. 48.5 ± 12.3 mmHg, *p* = 0.001). Finally, in terms of MetS features, the elevated waist circumference (*p* = 0.002) and decreased HDL cholesterol level (*p* = 0.040) criteria were met more frequently in women fasters, while the elevated blood pressure status was significantly more frequent in men (*p* < 0.001). However, the presence of MetS did not differ significantly between genders (*p* = 0.488).

In the group of non-fasters, men had significantly higher weight (85.5 ± 14 vs. 66.6 ± 12 kg, *p* < 0.001), height (1.76 ± 0.07 vs. 1.62 ± 0.05 cm, *p* < 0.001), BMI (27.2 ± 3.6 vs. 25.3 ± 4.7 kg/m^2^, *p* < 0.002), fat free mass (63.5 ± 7.78 vs. 43.5 ± 3.4 kg, *p* < 0.001), waist circumference (94.6 ± 112 vs. 84.9 ± 12.8 cm, *p* < 0.001), and waist-to-hip ratio (0.95 ± 0.1 vs. 0.86 ± 0.1, *p* < 0.001), while women had significantly greater mean body fat percentage (33.3 ± 8.2 vs. 25 ± 7.5%, *p* < 0.001). Mean SBP and DBP were higher in men versus women (SBP 135.7 ± 11.4 vs. 124.6 ± 13.9 mmHg, *p* < 0.001, and DBP 84.0 ± 8.4 vs. 78.0 ± 8.8 mmHg, *p* < 0.001, respectively).

Regarding the macro- and micro-nutrient intake, men reported significantly greater intakes of energy (1830 ± 500 vs. 1564 ± 440 kcal, *p* < 0.001), protein (67.5 ± 22.5 vs. 54.2 ± 18.7 g, *p* < 0.001), carbohydrates (177.2 ± 64.8 vs. 154.3 ± 51.6 g, *p* = 0.007), dietary fiber (23.3 ± 11.8 ± 20 ± 9 g, *p* = 0.031), other carbohydrates (78.6 ± 34.2 vs. 69 ± 27.7 g, *p* = 0.031), fat (95 ± 28.2 vs. 85 ± 28.3 g, *p* = 0.016), saturated (27.5 ± 10.8 vs. 23.2 ± 9.16 g, *p* = 0.003) and monounsaturated fat (47 ± 15 vs. 42 ± 15 g, *p* = 0.015), and water (824 ± 303 vs. 637 ± 223 mL, *p* < 0.001) compared with women. In turn, women had significantly higher mean values of HDL cholesterol (59 ± 16 vs. 47 ± 11.5 mmHg, *p* < 0.001) and phosphorus (5.8 ± 1.3 vs. 5.4 ± 1.2 mg/dL, *p* = 0.028), and lower mean values of iron (88.7 ± 38.5 vs. 106.8 ± 40.2 μg/dL, *p* = 0.002), creatinine (0.9 ± 0.1 vs. 1.1 ± 0.2 mg/dL, *p* < 0.001), urea (29.6 ± 9.3 vs. 33.6 ± 10.7 mg/dL, *p* = 0.006), uric acid (4.1 ± 1.1 vs. 5.3 ± 1.2 mg/dL, *p* < 0.001), SGOT (13.7 ± 8 vs. 16.6 ± 9.5 U/L, *p* = 0.021), SGPT (9.34 ± 8.6 vs. 12.3 ± 9.2 U/L, *p* = 0.025), and magnesium (1.7 ± 0.2 vs. 1.8 ± 0.2 mg/dL, *p* = 0.029). Some of the MetS components were observed significantly more frequently in men, i.e., elevated triglycerides (*p* = 0.040) and elevated BP (*p* < 0.001), whereas MetS prevalence was higher in men with a marginal level of significance (*p* = 0.050).

Analysis was further carried out on the basis of gender between the groups, in order to investigate any potential impact of diet status. Analysis in men showed that non-fasters had significantly increased systolic (*p* = 0.015) and diastolic pressure (*p* = 0.046). In relation to the nutrient intake, non-fasters had an almost significant increased consumption of calories (*p* = 0.051), significantly lower intake of protein (*p* = 0.010), higher total sugars (*p* = 0.020), higher cholesterol (*p* = 0.049), and higher alcohol consumption (*p* = 0.009). As far as blood analysis is concerned, non-fasters had statistically higher urea (*p* = 0.007) and lower magnesium (*p* = 0.032). On the other hand, for the female group, we noticed the following. With regard to anthropometric data, non-fasters had statistically higher diastolic blood pressure (*p* = 0.025) and pulses (*p* = 0.006), and lower BMI (*p* = 0.043) and fat free mass (*p* = 0.016). Statistically lower intake of protein (*p* = 0.027), alongside statistically higher intake of fat (*p* = 0.025), saturated fat (*p* = 0.006), and cholesterol (*p* = 0.014), was noticed in non-fasters. Last, statistically higher levels of albumin (*p* = 0.001), SGOT (*p* = 0.023), SGPT (*p* = 0.019), phosphorus (*p* < 0.001), and glucose (*p* = 0.038), alongside statistically lower insulin (*p* = 0.004) and magnesium (*p* < 0.001), were found. All results can be found in Tables S2–S4.

## 4. Discussion

To the best of our knowledge, this is the first study that has focused on the impact of COC fasting on body composition and MetS in a COC fasting population in Greece. Fasters were found to follow healthier lifestyle habits, i.e., less frequently reporting smoking, alcohol intake, anxious way of eating, use of diet products, watching TV, and spending time in front of screens (mobile and PC) compared with non-fasters. These findings are in agreement with the study of Chliaoutakis et al., showing that, among a sample of 250 healthy adults aged 20 to 65 years, those who followed the COC fasting recommendations (*n* = 79) adopted healthier lifestyle habits, i.e., abstaining from tobacco and alcohol use, as well as being more physically active [25]. Moreover, Sarri et al. reported abstinence from smoking in 60 fasters compared with 60 non-fasters [26]. The COC supports a positive healthy behavior, in terms of nutrition as well as lifestyle habits, including abstinence from smoking and moderate alcohol consumption, as wine is allowed during some days. This seems to also have a positive effect on the general quality of life of fasters [25]. According to the literature, statistically higher alcohol intake was noticed in non-faster men vs. faster men. Furthermore, as part of a healthier lifestyle, in our study, it was found that fasters were active 3–5 times per week and/or everyday (16.5% in total) in comparison to non-fasters (13% in total), but the difference was not significant. No other study focusing on the COC fasting has investigated and/or reported this aspect, according to our knowledge.

In the present study, no significant differences between the two study groups were found in terms of anthropometric characteristics, except for DBP, being significantly lower in fasters vs. non-fasters. Limited evidence exists for the impact of COC fasting to blood pressure. Similar to our study, El-Sayed et al. found that 49 fasters decreased their DBP after a fasting period [18], and Papadaki et al. reported that fasters had lower DBP compared with non-fasters during a non-fasting period [27]. Statistical difference was also noticed when we further sub-analyzed our population on the basis of gender. In men, statistically higher systolic and diastolic blood pressure was found in non-fasters compared to fasters. Moreover, in women, non-fasters had statistically higher diastolic blood pressure.

Although no significant difference was found in the BMI, it is worth discussing the high mean BMI in both fasters and non-fasters. The high BMI mean values (≥25 kg/m^2^) could be a result of the low physical activity that was reported by the majority of fasters and non-fasters, with a few never being active at all. High BMI values were also reported in 37 fasters and 48 non-fasters that were followed for a 40-days fasting period, in both fasting and non-fasting periods [26]. With further analysis, on the basis of the gender between the groups, it was seen that non-faster women had statistically lower BMI and fat free mass. This could be a result of diet, physical activity level, age, and hormonal changes driven by the postmenopausal status of women, as well as influenced by any other lifestyle habit, and it is an interesting area to further explore.

Overall, significant differences were observed in nutrient intake and biochemistry data. For example, COC fasters had less energy intake than non-fasters during a non-fasting period. Other studies also reported reduced energy intake, ranging from −10% in Greek fasters (*n* = 60) compared with non-fasters (*n* = 60) during a fasting period [13,14,24] up to −20% observed among 36 during a fasting period [20]. Another Greek study, including 43 general population male fasters and 57 male monks, demonstrated a reduction in the energy intake of the fasters during a fasting period [21]. Furthermore, in a sample of 99 COC fasters from the USA, energy intake among fasters was significantly lowered by a mean of 113 kcals/day [28]. These findings support that energy intake is lower in fasters compared with non-fasters during the fasting periods. However, the results of the present study are even more important, since the sample size was higher (i.e., 400 participants) and refers to a non-fasting period, thus highlighting that fasters tend to adopt healthier dietary habits in their daily life (i.e., even in non-fasting periods). Of note, apart from lower energy intake, in the present study, fasters consumed significantly less protein; fat; saturated, monounsaturated, and polyunsaturated fatty acids; and cholesterol compared with non-fasters during this non-fasting period. Sarri et al. reported less intake of protein, fat, saturated fatty acids, and cholesterol in fasters versus non-fasters during a fasting period [15,26,29]. To continue, when analyzed on the basis of gender, a statistically lower intake of total fat and saturated fat were noticed in non-faster women, and a lower intake of cholesterol in both non-faster women and men.

With regard to biochemical parameters, non-fasters had significantly higher levels of urea, transaminases, phosphorus, and glucose, whereas they had significantly lower levels of insulin and magnesium compared with fasters. The clinical significance of these findings should be further evaluated. According to our knowledge, no other published study has focused on these blood parameters. Differences in biochemical data should be further analyzed with a focus on the food intake and dietary pattern analysis.

Noteworthy, fasters were significantly less likely to have two of the MetS components, i.e., hyperglycemia and hypertension, as well as less likely to develop MetS (although this latter finding did not achieve statistical significance). This finding is of great importance, since MetS has been linked to an increased risk of developing several cardiometabolic diseases [30,31,32,33,34,35].

Furthermore, according to the literature, there is no other study focusing on the effects of COC fasting on MetS prevalence, since other studies examined only MetS individual components. For example, in a study conducted in Egypt with 49 fasters and 48 non-fasters, after a fasting period of 48 days, both systolic and DBP were decreased in COC fasters with type 2 diabetes [18]. Similar results were reported in Greece during COC fasting periods in both monks [27] and people of the general population [36]. Furthermore, with regard to fasting blood glucose, it was shown that in 36 healthy fasters, after a 43-day fasting period, glucose levels remained stable [19]. Similarly, no statistically significant change was detected in fasting glucose in 49 fasters with type 2 diabetes mellitus after a 48-day fasting period [18].

In the present study, gender subgroup analysis was also performed. Overall, men had higher mean weight, height, fat free mass, waist circumference, and waist-to-hip ratio, whereas women had higher mean body fat and lower BP, in both the fasting and non-fasting groups. To the best of our knowledge, no other study has evaluated gender differences in relation to these anthropometric data. Regarding the macro- and micro-nutrient intake, both faster and non-faster men had significantly greater mean intake of energy, protein, other carbohydrates, total fat, polyunsaturated and monounsaturated fat, and water compared with women. Relevant data are limited. Of note, during a 48-day fasting period, 42 U.S. male fasters consumed significantly less energy comparing to 57 females [28]. Additionally, in the present study, gender differences in biochemical parameters were also observed. For example, women had significantly higher mean HDL cholesterol values compared with men in both the fasting and non-fasting groups. Similarly to our study, Sarri et al. showed that women (*n* = 29) who fasted according to the COC had higher HDL levels than men (*n* = 31) [15]. Finally, MetS prevalence was marginally greater in non-fasting men compared with women.

The present study has some limitations, starting with selection bias, as all subjects were volunteers, and there was no possible way to examine the full adherence to COC fasting, apart from the participants’ self-report; moreover, the majority of participants were women, and we are unsure about the effects of gender on the outcomes. Furthermore, the macro- and micro-nutrient intake was based on self-reporting questionnaires with the use of the 24 h dietary recall and FFQ, thus potentially leading to under- or over-reporting of food intakes. However, it should be noted that the present study involved one of the largest groups of COC fasters in the literature so far (i.e., 200) [3,37]. Moreover, the present findings refer to a non-fasting period, thus representing daily life. Other strengths of this study include the results on MetS prevalence and gender analysis.

## 5. Conclusions

In the present study, during a non-fasting period, individuals following the COC fasting recommendations reported lower intake of calories, protein, fat, and cholesterol compared with non-fasters. Furthermore, non-fasters were significantly more likely to have hyperglycemia and hypertension, leading to a non-significant higher rate of MetS prevalence compared with fasters. Other biochemical factors significantly differed between the two study groups. Overall, the results of the present study highlighted the adoption of a healthier lifestyle by fasters (vs. non-fasters), even in non-fasting periods. Further research is warranted to establish the long-term clinical impact of these findings.

## Figures and Tables

**Table 1:** Anthropometric parameters of the two study groups.

Variable	Fasters (*n* = 200)	Non-Fasters (*n* = 200)	*p*-Value
	Mean ± SD	Mean ± SD	
SBP (mmHg)	126 ± 13	129 ± 14	0.054
DBP (mmHg)	78 ± 9	80 ± 9	0.003
Pulses (per minute)	69 ± 10	71 ± 10	0.055
Weight (kg)	74.4 ± 15.4	73.6 ± 15.5	0.63
Height (m)	1.7 ± 0.1	1.7 ± 0.1	0.12
BMI (kg/m^2^)	26.7 ± 4.7	26.0 ± 4.4	0.11
Body fat (%)	30.8 ± 9.7	30.3 ± 8.9	0.53
Body fat (kg)	23.6 ± 10.4	22.7 ± 9.2	0.34
Fat free mass (kg)	50.9 ± 10.0	50.9 ± 11.1	0.95
Waist circumference (cm)	89.3 ± 14.3	88.5 ± 13.2	0.55
Hip circumference (cm)	99.3 ± 9.3	98.4 ± 7.4	0.31
WHR	0.9 ± 0.1	0.9 ± 0.1	0.89

SBP = systolic blood pressure, DBP = diastolic blood pressure, BMI = body mass index, WHR = waist-to-hip ratio, SD = standard deviation.

**Table 2:** Demographics, lifestyle, and socioeconomic habits of the two study groups.

Variable	Fasters (*n* = 200)		Non-Fasters (*n* = 200)		*p*-Value
	*n*	%	*n*	%	
**Sex**					0.60
Male	69	34.5	74	37.0	
Female	131	65.5	126	63.0	
**Education level**					0.17
Primary education	9	4.5	17	8.5	
Secondary education	51	25.5	54	27.0	
Tertiary education	113	56.5	104	52.0	
Master’s/doctoral	27	13.5	25	12.5	
**Marital status**					0.67
Single	88	44.0	94	47.0	
Married/living together	108	54.0	96	48.0	
Divorced	4	2.0	5	2.5	
Widowed	-		5	2.5	
**Smoking status**					0.000
Yes	11	5.5	58	71.0	
No—never	183	91.5	142	29.0	
No—quit smoking	6	3.0	-		
**Body mass index status**					0.15
Underweight (<18.5 kg/m^2^)	-		2	1.0	
Normal weight (18.5–24.9 kg/m^2^)	79	39.5	91	45.5	
Overweight (25–29.9 kg/m^2^)	76	38.0	68	34.0	
Obesity (≥30 kg/m^2^)	45	22.5	39	19.5	
**Supplement status**					0.71
Yes	43	21.5	40	20.0	
No	157	78.5	160	80.0	
**Way of eating**					0.004
Anxious	7	3.5	4	2.0	
Slowly	77	38.5	98	49.0	
Quickly	75	37.5	85	42.5	
Regular	41	20.5	13	6.5	
**Way of eating—company**					0.11
Sitting alone	48	24.0	47	23.5	
Sitting with someone	145	42.5	130	65.0	
In front of a TV/computer	7	3.5	23	11.5	
**Way of cooking**					0.47
Boiled	51	25.5	46	23.0	
Fried	117	58.5	122	61.0	
Grilled/oven	23	11.5	29	14.5	
Mixed methods	4	2.0	3	1.5	
**Use of salt apart of cooking process**					0.17
Yes	58	29.0	46	23.0	
No	142	71.0	154	77.0	
Use of diet products					0.002
Yes	36	18.0	62	31.0	
No	164	82.0	138	69.0	
**Physical activity level/status**					0.06
Extremely low *(never/rarely)*	12	6.0	27	13.5	
Low *(<2 times per week)*	57	28.5	53	26.5	
Moderate *(2–3 times per week)*	98	49.0	94	47.0	
High (*3–5 times per week)*	30	15.0	22	11.0	
Extremely high *(everyday)*	3	1.5	4	2.0	
**Free-time workout**					0.26
Yes	74	37.0	85	42.5	
No	126	63.0	115	57.5	
	**Mean ± SD**		**Mean ± SD**		***p*-Value**
**Age (years)**	43.4 ± 16.7		41.9 ± 17.3		0.40
**Frequency of free-time workouts (times/week)**	3.4 ± 2.0		4.1 ± 1.9		0.021
**Duration of free-time workouts (hours/activity)**	1.2 ± 0.7		1.2 ± 0.7		0.81
**Total duration of free-time workouts (hours/week)**	1.6 ± 3.4		2.1 ± 3.5		0.18
**Sleeping (hours/night)**	6.8 ± 1.2		6.9 ± 1.2		0.23
**Sleeping (hours/day)**	0.8 ± 0.9		1.2 ± 1.4		0.008
**Watching TV (hours/day)**	1.1 ± 1.3		2.2 ± 1.7		0.000
**Screen time (PC/mobile) (hours/day)**	2.3 ± 2.9		2.9 ± 2.9		0.043
**Reading (hours/day)**	1.4 ± 1.2		1.3 ± 1.4		0.16

SD = standard deviation.

**Table 3:** Nutrient intake of the two study groups.

**Variable**	**Fasters (*n* = 200)**	**Non-Fasters (*n* = 200)**	***p*-Value**
	**Mean ± SD**	**Mean ± SD**	
Energy (kcal)	1547 ± 389	1662 ± 480	0.009
Protein (g)	52.2 ± 47.5	59.1 ± 21.1	0.001
Carbohydrates (g)	159.9 ± 48.5	162.8 ± 57.8	0.59
Dietary fiber (g)	20.6 ± 7.8	21.2 ± 10.2	0.18
Soluble fiber (g)	2.0 ± 1.2	2.0 ± 1.6	0.99
Sugar total (g)	47.5 ± 24.6	51.4 ± 51.4	0.13
Monosaccharides (g)	16.8 ± 10.9	15.1 ± 12.8	0.15
Disaccharides (g)	13.4 ± 10.4	15.3 ± 15.5	0.06
Other carbs (g)	69.6 ± 26.1	72.5 ± 66.7	0.31
Fat (g)	81.6 ± 27.4	88.7 ± 85.9	0.012
Saturated fat (g)	21.5 ± 9.6	24.8 ± 22.4	0.001
Monounsaturated fat (g)	42.0 ± 16.1	43.7 ± 43.8	0.030
Polyunsaturated fat (g)	8.9 ± 3.4	9.9 ± 9.3	0.009
Trans fatty acids (g)	0.7 ± 0.7	0.9 ± 0.5	0.08
Cholesterol (mg)	147 ± 97	178 ± 159	0.001
ω-3 fatty acids (mg)	0.7 ± 0.4	0.7 ± 0.6	0.73
ω-6 fatty acids (mg)	5.1 ± 2.9	5.5 ± 4.5	0.27
Water (L)	710 ± 228	706 ± 654	0.86
Alcohol (g)	1.6 ± 4.7	4.1 ± 0.0	0.002

SD = standard deviation.

**Table 4:** Blood analysis.

Variable	Fasters (*n* = 200)	Non-Fasters (*n* = 200)	*p*-Value
	Mean ± SD	Mean ± SD	
Fe (μg/dL)	99 ± 38	95 ± 40	0.29
Creatinine (mg/dL)	0.93 ± 0.16	0.96 ± 0.17	0.069
Urea (mg/dL)	29 ± 12	31 ± 10	0.049
Uric acid (mg/dL)	4.4 ± 1.3	4.6 ± 1.3	0.41
Albumin (g/dL)	4.35 ± 0.26	4.40 ± 0.29	0.09
γ-GT (U/L)	20 ± 13	22 ± 38	0.33
Ca (mg/dL)	9.8 ± 0.6	9.8 ± 0.6	0.54
ALP (U/L)	66 ± 20	70 ± 29	0.10
Total cholesterol (mg/dL)	188 ± 46	196 ± 49	0.09
Triglycerides (mg/dL)	133 ± 91	144 ± 91	0.25
HDL (mg/dL)	54 ± 16	54 ± 15	0.62
LDL (mg/dL)	115 ± 68	110 ± 53	0.37
SGOT (U/L)	13 ± 7	15 ± 9	0.016
SGPT (U/L)	8 ± 7	10 ± 9	0.008
Vitamin D (ng/mL)	18 ± 7	19 ± 6	0.38
Folic acid (ng/mL)	3.5 ± 4.3	3.3 ± 5.6	0.64
Vitamin B12 (pg/mL)	337 ± 280	340 ± 139	0.89
Insulin (μIU/m)	5.4 ± 15.5	3.1 ± 2.6	0.036
Magnesium (mg/dL)	1.9 ± 0.2	1.8 ± 0.2	<0.001
CRP (mg/dL)	0.2 ± 0.2	0.2 ± 0.3	0.87
Phosphorus (mg/dL)	5.0 ± 1.6	5.7 ± 1.3	<0.001
Glucose (mg/dL)	82 ± 14	86 ± 21	0.018

ALP = alkaline phosphatase, HDL = high-density lipoprotein, LDL = low-density lipoprotein, SGOT = serum glutamic oxaloacetic transaminase, SGPT = serum glutamic pyruvic transaminase, CRP = C-reactive protein.

**Table 5:** Prevalence of metabolic syndrome and its components.

Variable	Fasters (*n* = 200)	Non-Fasters (*n* = 200)	*p*-Value
	*n* (%)	*n* (%)	
WC > 102 cm for men or >88 cm for women	81 (40.5)	68 (34)	0.18
FBG ≥ 100 mg/dL	13 (6.5)	28 (14.0)	0.013
HDL cholesterol < 40 mg/dL for men or <50 mg/dL for women	62 (31.0)	56 (28)	0.51
TRG ≥ 150 mg/dL	63 (31.5)	71 (35.5)	0.39
BP ≥ 130/85 mmHg	34 (17.0)	67 (33.5)	<0.001
Metabolic syndrome prevalence	31 (15.5)	42 (21.0)	0.15

WC: waist circumference, FBG: fasting blood glucose, HDL: high-density lipoprotein, TRG: triglycerides, BP: blood pressure.

## Data Availability

The raw data supporting the conclusions of this study are available from the corresponding author upon request.

## Supplementary Materials

The following supporting information can be downloaded at: https://www.mdpi.com/article/10.3390/nu15112468/s1, Table S1: Medians and percentiles of demographics and lifestyle habits of the two study groups; Table S2: Anthropometric analysis based on gender; Table S3: Nutrient intake analysis based on gender; Table S4: Blood analysis based on gender.
