# Supplementary material for: Nutrient Intake and Risk Factors for Metabolic Syndrome in Christian Orthodox Church Religious Fasters
Source: Nutrients. 2023 May 25;15(11):2468. doi: 10.3390/nu15112468 (PMC10255146; doi:10.3390/nu15112468)
Supplement: Supplementary file 1 [file nutrients-15-02468-s001.zip › nutrients-2390664-supplementary.pdf]

Supplemental material

Supplemental Table 1. Medians and percentiles of demographics and lifestyle habits of the two study groups.

Variables	Fasters (n=200)				Non-fasters (n=200)			
	Median	Percentile 25%	Percentile 50%	Percentile 75%	Median	Percentile 25%	Percentile 50%	Percentile 75%
Age (years)	44.7	26.7	44.7	58.2	46.1	23.9	46.1	56.4
Frequency of free time workouts (times/week)	3	2	3	5	4	3	4	5
Duration of free time workouts (hours/activity)	1	0..8	1	1.5	1	1	1	1.5
Total duration of free time workouts (hours/week)	0	0	0	2.3	0	0	0	3
Sleeping (hours/night)	7	6	7	8	7	6	7	8
Sleeping (hours/day)	1	0	1	1	1	0	1	2
Watching TV (hours/day)	1	0	1	2	2	1	2	3
Screen time (PC/mobile) (hours/day)	1	0	1	3.1	2	0.5	2	4
Reading (hours/day)	1	1	1	2	1	0	1	2

Supplemental Table 2. Anthropometric analysis based on gender

Variable	Men (n=143)		p-value	Women (n=257)		p-value
	Fasters	Non-fasters		Fasters	Non-fasters	
	Mean ± SD			Mean ± SD		
SBP (mmHg)	130 ± 12	135 ± 11	0.015	123.5 ± 13.5	124.6 ± 13.8	0.51
DBP (mmHg)	81 ± 8	84 ± 8	0.046	75.6 ± 8.3	78 ± 8.8	0.025
Pulses (per minute)	69 ± 10	69 ± 11	0.89	69.5 ± 8.9	72.7 ± 9.5	0.006
Weight (kg)	83.5 ± 16	85.5 ± 14	0.43	69.5 ± 12.5	66.6 ± 11.7	0.055
Height (m)	174 ± 0.06	176 ± 0.07	0.07	162 ± 0.05	162 ± 0.05	0.54
BMI (kg/m^2)	27.2 ± 4.8	27.2 ± 3.6	0.99	26.5 ± 4.7	25.2 ± 4.7	0.043
Body fat (%)	23.8 ± 8.2	25 ± 7.4	0.36	34.6 ± 8.3	33.3 ± 8.2	0.22

**Body fat (kg)**	21 ± 9	22 ± 9	0.53	25 ± 9.9	23 ± 9.5	0.10
**Fat free mass (kg)**	62.5 ± 7.3	63.5 ± 7.7	0.45	44.6 ± 3.6	43.5 ± 3.4	0.016
**Waist circumference (cm)**	92.5 ± 14.2	94.6 ± 11.3	0.33	87.6 ± 14	85 ± 13	0.10
**Hip circumference (cm)**	97.9 ± 7.5	98.9 ± 6.4	0.38	100 ± 10	98 ± 8	0.09
**WHR**	0.94 ± 0.1	0.95 ± 0.1	0.41	0.87 ± 0.11	0.86 ± 0.10	0.33

**SBP=Systolic Blood Pressure, DBP=Diastolic Blood Pressure, BMI=Body Mass Index, WHR=Waist to Hip Ratio, SD=standard deviation.*

Supplemental Table 3. Nutrient intake analysis based on gender

**Variable**	**Men (n=143)**		**p-value**	**Women (n=257)**		**p-value**
	**Fasters**	**Non-fasters**		**Fasters**	**Non-fasters**	
	**Mean ± SD**			**Mean ± SD**		
**Energy (kcal)**	1677 ± 413	1829 ± 500	0.051	1479 ± 358	1563 ± 440	0.09
**Protein (g)**	87.8 ± 21.6	67.5 ± 22.4	0.010	49.3 ± 16.7	54.2 ± 18.7	0.027
**Carbohydrates (g)**	167.6 ± 51.6	177.2 ± 64.8	0.33	156 ± 45	154 ± 51	0.79
**Dietary fiber (g)**	22 ± 8.2	23.2 ± 11.7	0.48	19.8 ± 7.5	20 ± 9	0.83
**Soluble fiber (g)**	2.1 ± 1.2	2 ± 1.4	0.59	1.94 ± 1.1	2 ± 1.4	0.71
**Sugar total (g)**	46 ± 21	56 ± 25	0.020	48.3 ± 26	48.8 ± 26.8	0.88
**Monosaccharides (g)**	17.7 ± 9.8	17 ± 10	0.69	16.3 ± 11	14.1 ± 11	0.11
**Disaccharides (g)**	12.5 ± 9	16 ± 10	0.048	14 ± 10	15 ± 10	0.39
**Other carbs (g)**	77 ± 25	78 ± 34	0.66	66 ± 25.7	69 ± 28	0.39
**Fat (g)**	90 ± 28	95 ± 28	0.27	77.2 ± 26.1	85 ± 28.3	0.025
**Saturated fat (g)**	24.2 ± 9.8	27.4 ± 10.8	0.06	20 ± 9	23.2 ± 9	0.006
**Monounsaturated fat (g)**	45.2 ± 17	74 ± 14.7	0.50	40.3 ± 15.4	41.6 ± 15.2	0.48
**Polyunsaturated fat (g)**	9.5 ± 3.4	10.7 ± 4.5	0.06	8.6 ± 3.3	9.5 ± 4.6	0.06
**Trans fatty acids (g)**	1 ± 0.3	1.5 ± 0.7	0.56	0.6 ± 0.07	0.9 ± 0.08	0.08
**Cholesterol (mg)**	162 ± 100	193 ± 86	0.049	140 ± 93	168 ± 94	0.014
**ω-3 fatty acids (mg)**	0.65 ± 0.3	0.74 ± 0.4	0.20	0.66 ± 0.05	0.64 ± 0.03	0.62
**ω-6 fatty acids (mg)**	5.46 ± 3.4	5.53 ± 4.1	0.90	4.95 ± 2.5	5.47 ± 3.8	0.19
**Water (ltr)**	754.3 ± 224	824 ± 303	0.123	687.2 ± 228	637 ± 223	0.07
**Alcohol (g)**	2.8 ± 5.8	7.7 ± 4.2	0.009	0.91 ± 0.5	1.9 ± 1	0.12

**SD=standard deviation.*

Supplemental Table 4. Blood analysis based on gender

Variable	Men (n=143)		p-value	Women (n=257)		p-value
	Fasters	Non-fasters		Fasters	Non-fasters	
	Mean ± SD			Mean ± SD		
**Fe (μg/dL)**	107.3 ± 40	107 ± 40	0.94	95.4 ± 36.2	88.7 ± 38.5	0.15
**Creatinine (mg/dL)**	1.02 ± 0.1	1.06 ± 0.1	0.07	0.88 ± 0.1	0.89 ± 0.1	0.37
**Urea (mg/dL)**	29.2 ± 8.2	33.6 ± 10.6	0.007	28.6 ± 14.3	29.6 ± 9.3	0.52
**Uric acid (mg/dL)**	5.3 ± 1.2	5.3 ± 1.2	0.95	4 ± 1	4.1 ± 1.1	0.38
**Albumin (g/dL)**	4.5 ± 0.2	4.4 ± 0.3	0.14	4.3 ± 0.2	4.4 ± 0.3	0.001
**γ-GT (U/l)**	24.4 ± 16	25.2 ± 14.6	0.76	17.1 ± 11.1	20.8 ± 17.1	0.38
**Ca (mg/dL)**	9.8 ± 0.4	9.8 ± 0.6	0.87	9.7 ± 0.7	9.8 ± 0.5	0.57
**ALP (U/l)**	70 ± 20	70.5 ± 23	0.83	64.6 ± 19	70.5 ± 32.3	0.08
**Total cholesterol (mg/dL)**	182.3 ± 38.5	188 ± 47.2	044	191 ± 49	201 ± 49	0.10
**Triglycerides (mg/dL)**	147 ± 103	152.2 ± 85.9	0.73	126 ± 84	138.7 ± 94.1	0.25
**HDL (mg/dL)**	49 ± 12	46 ± 11.5	0.37	56.3 ± 16.5	59 ± 15.8	0.20
**LDL (mg/dL)**	109.6 ± 61.4	105.3 ± 48.7	0.64	118 ± 71	112 ± 55	0.47
**SGOT (U/L)**	15 ± 9	16.6 ± 9.4	0.29	11.7 ± 5.9	13.6 ± 4.9	0.023
**SGPT (U/L)**	10.2 ± 9	12.3 ± 9.2	0.19	7.3 ± 5	9.4 ± 2.6	0.019
**Vitamin D (ng/ml)**	19 ± 7.4	19.6 ± 5.5	0.58	18 ± 7.2	18.5 ± 6.5	0.52
**Folic acid (ng/ml)**	3.7 ± 2	3 ± 2.1	0.31	3 ± 1.1	3.5 ± 1.4	0.86
**Vitamin B12 (pg/ml)**	301.6 ± 150	326.5 ± 126	0.28	356 ± 127.7	348 ± 146	0.80
**Insulin (μIU/m)**	7 ± 2.5	3.3 ± 2.7	0.21	4.5 ± 0.8	3 ± 2.5	0.004
**Magnesium (mg/dL)**	1.9 ± 0.2	1.8 ± 0.2	0.032	1.9 ± 0.2	1.76 ± 0.2	0.000
**CRP (mg/dL)**	0.2 ± 0.01	0.2 ± 0.03	0.44	0.2 ± 0.02	0.2 ± 0.03	0.68
**Phosphorus (mg/dL)**	4.9 ± 1.6	5.4 ± 1.2	0.029	5.1 ± 1.6	5.8 ± 1.3	0.000
**Glucose (mg/dL)**	84 ± 12	86.8 ± 14	0.27	81 ± 15	86 ± 22	0.038

**ALP=Alkaline Phosphatase, HDL=High-Density Lipoprotein, LDL=Low-Density Lipoprotein, SGOT=Serum Glutamic Oxaloacetic Transaminase, 0.79SGPT=Serum Glutamic Pyruvic Transaminase, CRP=C-reactive protein.*
